# Conditional survival analysis of patients with resected non–small cell lung cancer

**DOI:** 10.1016/j.xjon.2023.09.010

**Published:** 2023-09-18

**Authors:** Talib Chaudhry, Vaishnavi Krishnan, Andrew E. Donaldson, Zachary M. Palmisano, Sanjib Basu, Nicole M. Geissen, Justin M. Karush, Gillian C. Alex, Jeffrey A. Borgia, Michael J. Liptay, Christopher W. Seder

**Affiliations:** a Department of Cardiovascular and Thoracic Surgery, Rush University Medical Center, Chicago, Ill; b Department of Pathology, Rush University Medical Center, Chicago, Ill; c Department of Anatomy and Cell Biology, Rush University Medical Center, Chicago, Ill

**Keywords:** non–small cell lung cancer, conditional survival, disease-free survival, prognosis

## Abstract

**Background:**

Conditional survival (CS) analyses provide an estimate of survival accounting for years already survived after treatment. We aim to evaluate the difference between actuarial and conditional survival in patients following lung resection for non–small cell lung cancer (NSCLC). In addition, CS analyses are used to examine whether prognosticators of survival change over time following surgery.

**Methods:**

Patients who underwent anatomic lung resection at a single institution for pathologic stage I-IIIA NSCLC between 2010 and 2021 were identified; those who underwent wedge resection for node-negative tumors ≤2 cm were also included. CS estimates were calculated as the probability of remaining disease-free after *x* years of nonrecurrence (CS_*x*_). Kaplan–Meier, log-rank, and Cox proportional hazard methods for examining CS were used for subgroup comparisons and assessing associations with baseline covariates.

**Results:**

Overall, 863 patients met the study inclusion criteria, with a median follow-up of 44.1 months. Conditional overall survival (OS) and disease-free survival (DFS) were greater than actuarial rates at all time points after surgery. At the time of resection, male sex (hazard ratio [HR], 1.33; 95% confidence interval [CI], 1.03 to 1.72; *P* = .032), tumor size >3 cm (HR, 1.17; 95% CI, 1.11-1.23; *P* < .001), node positivity (HR, 3.31; 95% CI, 2.52-4.33; *P* < .001), and American Joint Committee on Cancer stage (*P* < .001) were associated with DFS. However, if a patient lived 3 years without recurrence (CS_3_), these factors were no longer prognostic of DFS.

**Conclusions:**

Conditional survival analyses provide dynamic assessments of OS and DFS after NSCLC resection. After 3 years without recurrence, certain characteristics associated with DFS at the time of surgery no longer prognosticate recurrence.

## Graphical abstract

**Figure:**
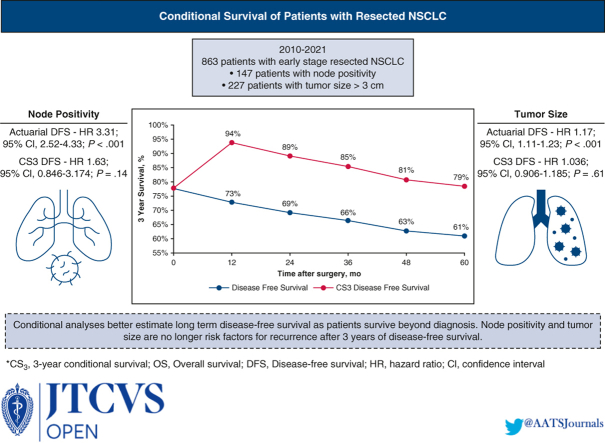

**Figure:**
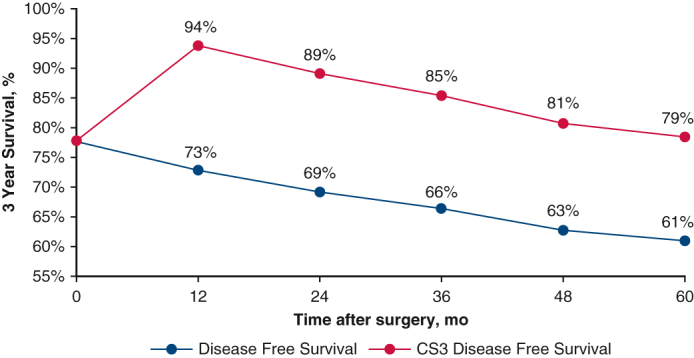
Disease free survival and 3-year conditional disease free survival of patients with resected non-small cell lung cancer. *CS3*, Conditional 3-year survival.

**Central Message**

As time progresses without recurrence, conditional survival analyses may better prognosticate survival than actuarial methods in patients with resected non–small cell lung cancer.

**Perspective**

Patients who continue to survive after cancer treatment experience dynamic changes to their risk for death and recurrence. Conditional survival analyses may allow for more accurate prognostication of these risks than actuarial estimates. The current data suggest an association between patient characteristics and overall and disease-free survival changes as patients live longer after treatment.

Lung cancer remains the leading cause of cancer-related deaths worldwide, with an estimated 127,000 deaths in the United States in 2023.^1^, ^2^, ^3^ However, recent advances in technology, screening, and therapeutics have resulted in earlier identification and more effective treatments, affecting traditional survival estimates.^4^, ^5^, ^6^ Prognosis varies across subtypes and staging, with better survival in patients with early-stage non–small cell lung cancer (NSCLC) than in those with more advanced stages. Treatment is primarily based on staging at the time of diagnosis with prognostication data available from that initial point in time. However, as patients survive beyond this, with dynamic changes in health, it becomes increasingly difficult to prognosticate overall survival (OS) and disease-free survival (DFS).^7^^,^^8^

Conditional survival (CS) analyses allow for estimation of survival with adjustment for the dynamic changes occurring after the initial diagnosis and as survivorship continues.^8^, ^9^, ^10^ Importantly, CS analyses account for years already survived after diagnosis, which can alter predictions of future survival.^11^ By understanding the CS of patients with lung cancer over time, providers may be able to individualize surveillance schedules and provide more accurate long-term prognostication.^11^

Although CS analyses have been reported for a variety of malignancies, the CS of patients with NSCLC has been largely performed internationally, with a paucity of data on DFS.^9^^,^^11^, ^12^, ^13^, ^14^, ^15^, ^16^, ^17^, ^18^, ^19^ In this study, we aimed to investigate the difference between actuarial and CS in patients following lung resection for NSCLC. In addition, we used CS analyses to examine whether prognosticators of survival change over time.

## Methods

Patients who underwent lung resection at Rush University Medical Center between 2010 and 2021 were identified from an institutional database. Inclusion criteria included patients with stage I-IIIA pathologically confirmed primary lung adenocarcinoma or squamous cell carcinoma who underwent anatomic lung resection with an R0 resection. Patients who underwent a wedge resection for a pathologic tumor ≤2 cm with N0 status were also included; the results of the CALGB14053 trial prompted this inclusion.^20^ Exclusion criteria included age <18 years, M1 disease, induction therapy, resection of multiple tumors, other nonlung primary malignancy, and <6 months of clinical follow-up from the date of surgery. The Institutional Review Board or an equivalent Ethics Committee of Rush University Medical Center approved the study protocol and publication of data (ORA 18070602, August 14, 2018, and ORA 18021402, June 14, 2018). The requirement for patient written consent for publication of the study data was waived owing to the study's retrospective nature.

Clinical data were collected on sex, race, ethnicity, Eastern Cooperative Oncology Group Performance Status, smoking status (current, ever, never), diabetes, coronary artery disease (CAD), congestive heart failure (CHF), histologic subtype, tumor size (largest measurement of invasive component; categorized as ≤3 cm vs >3 cm), node status, and American Joint Committee on Cancer (AJCC) eighth edition stage. Operative data included surgical procedure (wedge resection, segmentectomy, lobectomy, bilobectomy, or pneumonectomy), and surgical procedure date.

Postoperative data collected included adjuvant therapy, recurrence, date of recurrence, last known date alive, and date of death. OS was defined as time from surgery to the date of death or the last known date alive, and DFS was defined as time from surgery to first recurrence. In cases without recurrence, DFS was calculated until the last known date alive or date of death.

## Statistical Analysis

Conditional survival, CS_*x*_(*t*), was calculated as the probability of survival, where *x* is the time (in years) that the patient has already survived and *t* is the additional time beyond *x* that they may survive. Kaplan–Meier, log-rank, and Cox proportional hazard methods for CS were used for subgroup comparisons and associations with baseline covariates.^21^ Hazard ratios (HRs) were calculated to express the prognostic power of each covariate. Variables considered included age, sex, race, ethnicity, Eastern Cooperative Oncology Group Performance Status, smoking status, diabetes, CAD, CHF, histologic subtype, tumor size, node status, and AJCC eighth edition stage.

## Results

### Demographics and Characteristics

Overall, 863 patients met inclusion criteria. The median age of the study population was 75 years (interquartile range [IQR], 69-81 years); 57% (n = 489 of 863) were female, and 80% (n = 692 of 863) identified as Caucasian. Of the 85% (n = 737 of 863) of patients with a smoking history, 20% (174 of 863) were current smokers. Adenocarcinoma was the most prevalent histologic subtype (77%; 661 of 863), and the median tumor size was 2.6 cm (IQR, 1.4-3.2 cm). Overall, 69.5% (n = 600 of 863) of the population had pathologic stage I disease, with node positivity in 17% (n = 147 of 863). Lobectomy was performed in 68% of the patients (n = 589 of 863), and segmentectomy or wedge resection was performed in the majority of the remaining cases (Table 1).

**Table 1:** Patient characteristics (N = 863)

Variable	Value
Age, y, median (IQR)	76 (69-81)
Male sex, n (%)	374 (43)
Race, n (%)	
Caucasian	692 (80)
Black	112 (13)
Asian	26 (3)
Other	33 (4)
ECOG Scale	
0	522 (60)
1	333 (39)
2+	8 (1)
Smoking status	
Never	126 (15)
Ever	563 (65)
Current	174 (20)
Comorbidities	
Diabetes	168 (19)
Coronary artery disease	165 (19)
Congestive heart failure	33 (4)
Histology	
Adenocarcinoma	661 (77)
Squamous cell carcinoma	202 (23)
Tumor size, median cm (IQR)	2.6 (1.4-3.2)
Tumor size	
0-3 cm	636 (74)
>3 cm	227 (26)
Node status	
Negative	716 (83)
Positive	147 (17)
AJCC Stage	
I	600 (70)
II	168 (19)
IIIA	95 (11)
Surgical procedure	
Lobectomy	589 (68)
Segmentectomy	29 (3)
Wedge	201 (24)
Bilobectomy or pneumonectomy	44 (5)
Adjuvant therapy	186 (22)

*IQR*, Interquartile range; *ECOG*, East Cooperative Oncology Group; *AJCC*, American Joint Committee on Cancer.

### Actuarial Survival Versus CS

**Figure E1:**
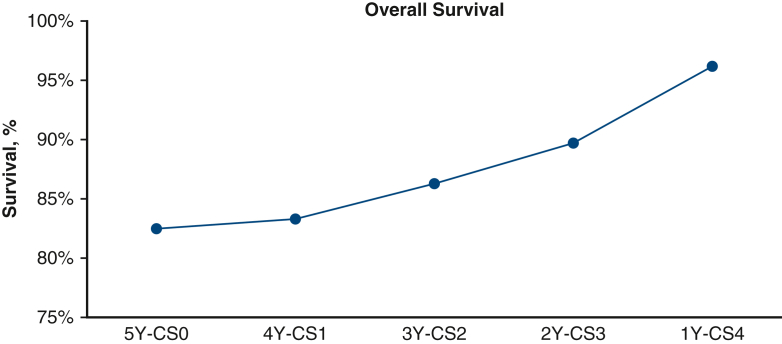
Overall survival by differing conditional survival. *CS*, Conditional survival.

**Figure E2:**
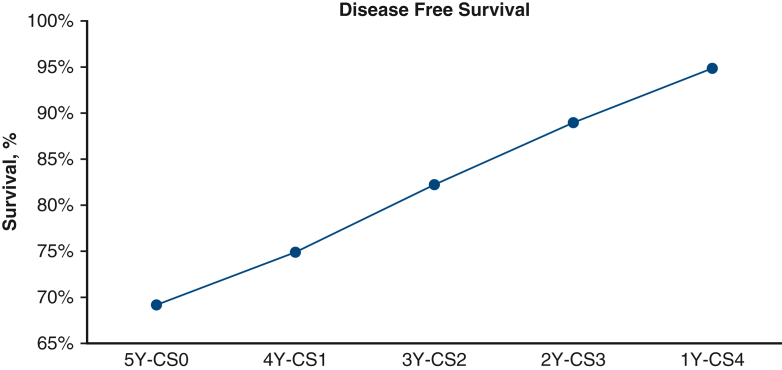
Disease-free survival by differing conditional survival. *CS*, Conditional survival.

The median follow-up was 44.1 months (IQR, 26.7-62 months) for OS and 38.2 months (IQR, 20.9-55.9 months) for DFS. After resection, actuarial OS was 99% at 1 year, 96% at 2 years, 92% at 3 years, and 83% at 5 years, and actuarial DFS was 92% at 1 year, 84% at 2 years, 78% at 3 years, and 69% at 5 years. A total of 132 patients, or 58% of our cohort, experienced a recurrence event within the first 2 years after surgery; 96 patients, or 42% of our cohort, experienced a recurrence event after the initial 2 years (Table E1). To fully evaluate the changes of survival estimates that conditional analyses would provide, we calculated the likelihood of surviving for up to 5 years after surgery; these were termed 5Y-CS_0_ consecutively through 1Y-CS_4_. For example, 2Y-CS_3_ corresponds to the probability of 3-year survival after already surviving 2 years. As patients survived longer after surgery, both their OS and DFS increased (Figures E1 and E2).

**Figure 1:**
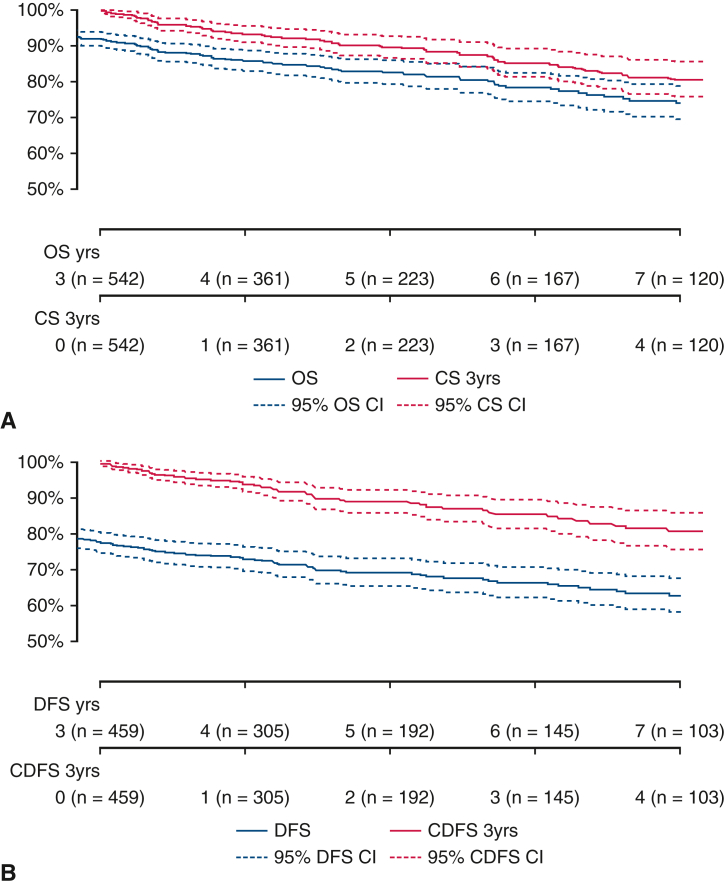
A, Kaplan–Meier plot comparing actuarial overall survival with 3-year conditional survival. B, Kaplan–Meier plot comparing actuarial disease-free survival with 3-year conditional disease-free survival. *OS*, Overall survival; *CS*, conditional survival; *DFS*, disease-free survival; *CDFS*, Conditional disease-free survival.

**Figure 2:**
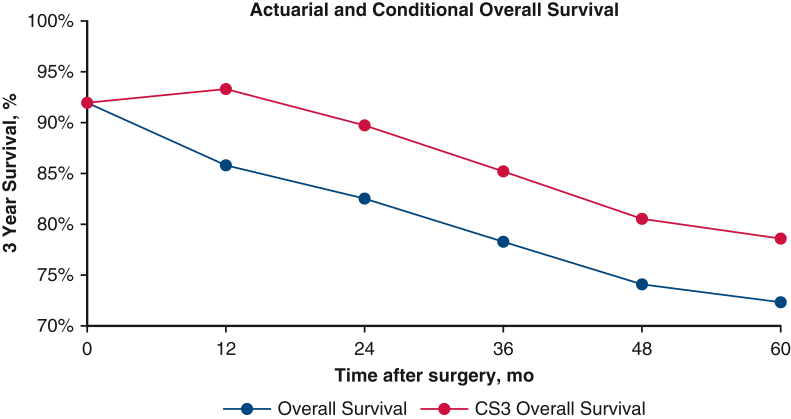
Comparisons of actuarial and 3-year conditional overall survival as time after surgery progresses. *CS3*, Conditional 3-year survival.

Conditional OS after 3 years of survival (CS_3_ OS) and conditional DFS after 3 years of nonrecurrence (CS_3_ DFS) were greater than their actuarial equivalents across all time points (Figure 1, *A* and *B*) Despite similar rates of change for both actuarial survival and CS, their absolute values for both CS_3_ DFS and OS were greater than their actuarial DFS and OS counterparts. For example, the CS_3_ OS at 2 years was 90%, whereas the actuarial OS at 5 years was 83%, and the CS_3_ DFS at 2 years was 89%, whereas the actuarial DFS at 5 years was 69%. Likewise, the CS_3_ OS at 5 years was 79%, whereas the actuarial OS at 8 years was 72% and the CS_3_ DFS at 5 years was 79% while the actuarial DFS at 8 years was 61% (Figure 2, *A* and *B*) The figures report the *y*-axis as 3-year survival; for example, at 24 months in the figure, the 3-year survival correlates with the 5-year survival, as discussed above.

### Characteristics Associated With Actuarial Survival

Clinicopathologic factors associated with actuarial OS included male sex (HR, 1.5; 95% CI, 1.06-2.14; *P* = .022), squamous cell histology (HR, 1.88; 95% CI, 1.23-2.71; *P* < .001), node positivity (HR, 2.46; 95% CI, 1.7-3.57; *P* < .001), tumor size >3 cm (HR, 1.13; 95% CI, 1.05-1.2; *P* < .001) and AJCC stage (*P* < .001). Similarly, factors associated with actuarial DFS included male sex (HR, 1.33; 95% CI, 1.02-1.72; *P* = .032), tumor size >3 cm (HR, 1.17; 95% CI, 1.11-1.23; *P* < .001), node positivity (HR, 3.31; 95% CI, 2.52-4.33; *P* < .001), and AJCC stage (*P* < .001).

### Characteristics Associated with CS at 3 Years (CS_3_)

Clinicopathologic factors associated with CS_3_ OS and CS_3_ DFS were examined. Similar to actuarial OS, squamous cell histology (HR, 1.86; 95% CI, 1.11-3.13; *P* = .017), node positivity (HR, 1.94; 95% CI, 1.14-3.31; *P* = .013), and AJCC stage (*P* < .001) were associated with CS_3_ OS. In addition, age >70 years was associated with CS_3_ OS (HR, 3.44; 95% CI, 1.25-9.46; *P* = .011). None of the factors associated with actuarial DFS, including male sex, tumor size >3 cm, node positivity, and AJCC stage, were associated with CS_3_ DFS.

Patients with characteristics most strongly associated with actuarial survival and recurrence at the time of surgery demonstrated the greatest difference between actuarial survival and CS. For example, node-positive patients demonstrated a Δ15% between actuarial 5-year OS (69%) and 2-year CS_3_ OS (84%) and Δ42% between actuarial 5-year DFS (40%) and 2-year CS_3_ DFS (82%). For comparison, node-negative patients showed only a Δ5% between actuarial 5-year OS (86%) and 2-year CS_3_ OS (91%) and Δ14% between actuarial 5-year DFS (76%) and 2-year CS_3_ DFS (90%).

**Figure E3:**
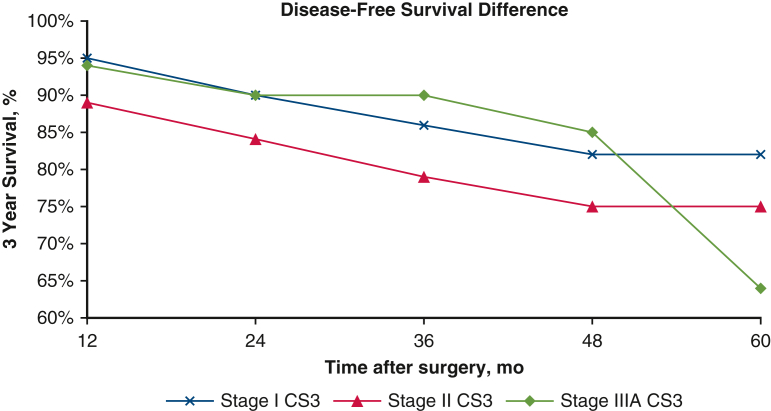
3-year conditional disease-free survival by stage. *CS3*, Conditional 3-year survival.

**Figure E4:**
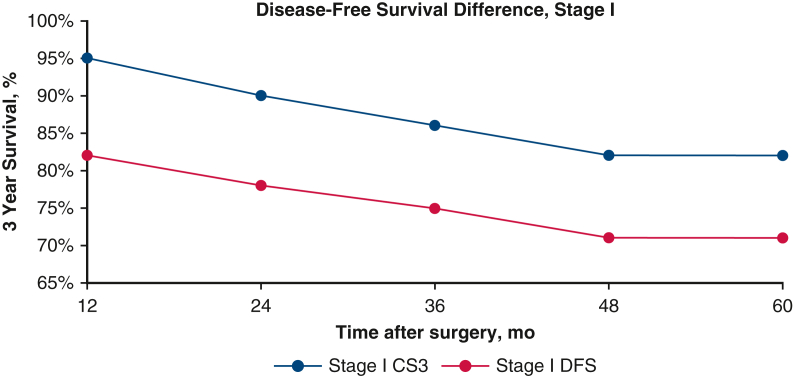
Comparison of disease-free survival (*DFS*) and CS3 DFS for stage I. *CS3*, Conditional 3-year survival.

**Figure E5:**
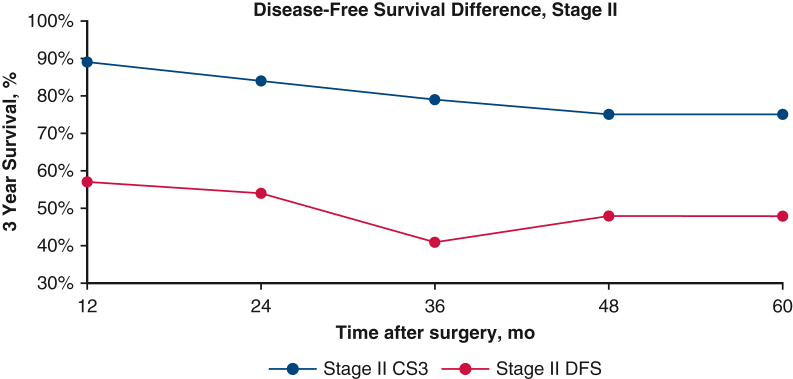
Comparison of disease-free survival (*DFS*) and CS3 DFS for stage II. *CS3*, Conditional 3-year survival.

**Figure E6:**
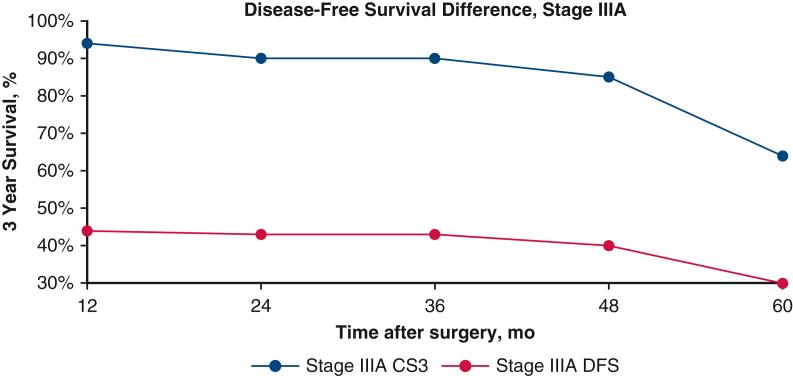
Comparison of disease-free survival (*DFS*) and CS3 DFS for stage IIIA. *CS3*, Conditional 3-year survival.

Similarly, patients with tumor size >3 cm had a Δ11% between actuarial 5-year OS (78%) and 2-year CS_3_ OS (89%) and a Δ29% between actuarial 5-year DFS (61%) and 2-year CS_3_ DFS (90%). Patients with tumor size ≤3 cm demonstrated Δ6% between actuarial 5-year OS (84%) and 2-year CS_3_ OS (90%) and Δ16% between actuarial 5-year DFS (72%) and 2-year CS_3_ DFS (88%). Likewise, patients with stage IIIA disease had a Δ19% between actuarial 5-year OS (65%) and 2-year CS_3_ OS (84%) and a Δ47% between actuarial 5-year DFS (43%) and a 2-year CS_3_ DFS (90%). For comparison, stage IA patients had only a Δ5% between actuarial 5-year OS (86%) and 2-year CS_3_ OS (91%) and a Δ12% between actuarial 5-year DFS (78%) and a 2-year CS_3_ DFS (90%) (Tables 2 and 3). Furthermore, CS_3_ DFS was greater than its actuarial equivalent when stratified by tumor stage (Figure E3, Figure E4, Figure E5, Figure E6).

**Table 2:** Difference between conditional survival and actuarial overall survival

Variable	Time after surgery			
	2 years	3 years	4 years	5 years
Male sex, %	8	9	8	7
Squamous cell cancer, %	11	10	9	7
Node positivity, %	15	14	12	12
Tumor >3 cm, %	11	10	9	8
AJCC stage III, %	19	18	17	14

*AJCC*, American Joint Committee on Cancer.

**Table 3:** Difference between conditional survival and actuarial disease-free survival

Variable	Time after surgery			
	2 years	3 years	4 years	5 years
Male sex, %	22	20	20	19
Squamous cell cancer, %	22	22	19	15
Node positivity, %	42	42	40	33
Tumor >3 cm, %	29	28	25	24
AJCC stage III, %	47	47	45	34

*AJCC*, American Joint Committee on Cancer.

**Figure 3:**
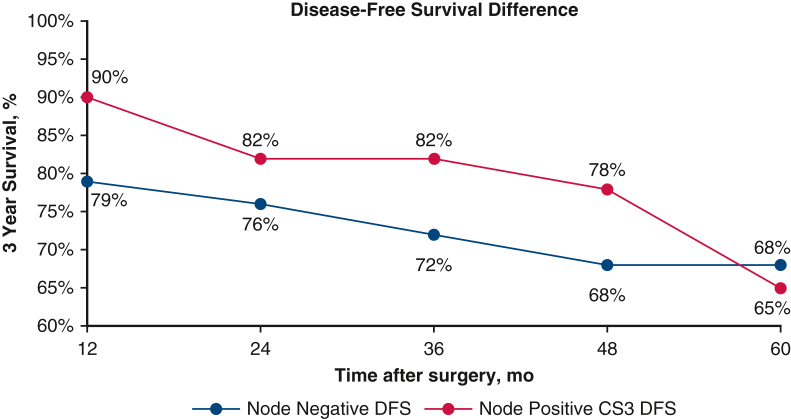
Comparison of the actuarial disease-free survival of node-negative patients versus the 3-year conditional disease-free survival of node-positive patients as time after surgery progresses. *DFS*, Disease-free survival; *CS*, conditional survival.

**Figure 4:**
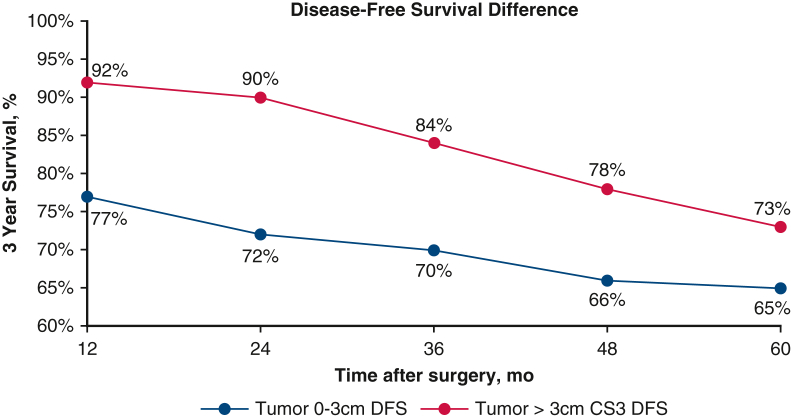
Comparison of the actuarial disease-free survival of patients with a tumor 0 to 3 cm versus the 3-year conditional disease-free survival of patients with a tumor >3 cm as time after surgery progresses. *DFS*, Disease-free survival; *CS*, conditional survival.

**Figure 5:**
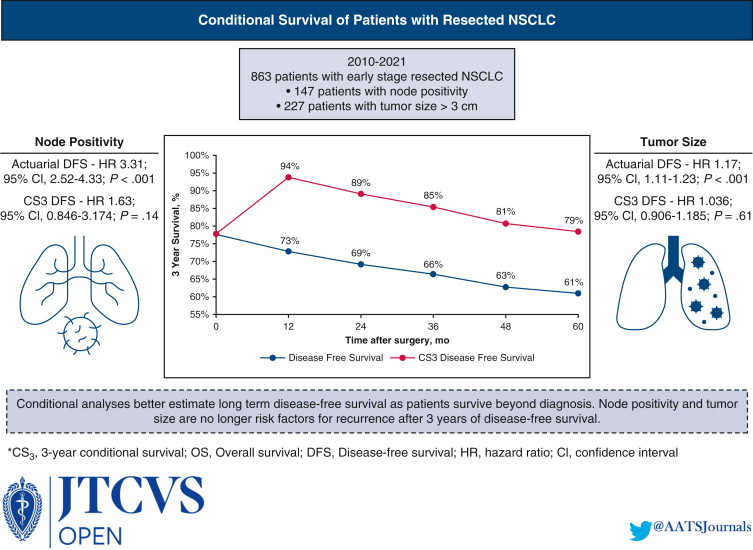
Graphical abstract representing key findings of this study.

In certain subgroups, patients with high-risk features at diagnosis had better CS estimates than the actuarial survival of patients without those features. For example, node-positive patients had a 2-year CS_3_ DFS of 82%,whereas node-negative patients had a 5-year actuarial DFS of 76%. Similarly, patients with a tumor size >3 cm had a 2-year CS_3_ DFS of 90%, whereas patients with a tumor size ≤3 cm had a 5-year actuarial DFS of 72%. This trend was observed across most time points following surgery (Figures 3 and 4). Figure 5 provides a graphical abstract of the study.

## Discussion

Traditional actuarial survival estimates take into account both early and late deaths, resulting in an overly pessimistic prognosis for patients who survive beyond the early postoperative period.^14^^,^^18^ The ability to prognosticate recurrence and survival from the current point in time rather than from estimates at the time of diagnosis can be helpful for both clinicians and patients. CS analyses provide information about how prognosis changes over time, as opposed to the static predictions provided by traditional survival estimates.^11^^,^^22^ Importantly, CS estimates allow for future survival estimates to be calculated as a dynamic function of time after treatment, but they do not account for perioperative events. Actuarial analyses include early postoperative mortalities, and when presented together with conditional estimates can provide a more comprehensive analysis by incorporating both the cumulative impact of events and the evolving nature of survival probabilities.

To our knowledge, this is the first North American study to compare actuarial and conditional OS and recurrence rates for patients who underwent curative intent lung resection for NSCLC. Our current data demonstrate that CS_3_ OS and CS_3_ DFS were greater than the actuarial rates at all time points after surgery. Patients with characteristics associated with mortality and recurrence at the time of resection exhibited the greatest differences between actuarial survival and CS. In addition, although male sex, tumor size >3 cm, node positivity, and AJCC stage were associated with DFS at the time of resection, after 3 years without recurrence, these factors no longer prognosticated DFS.

There is a relative paucity of CS analyses examining NSCLC in the literature. The current results are supported by a smaller, international study that found no association between sex, T stage, node positivity, or AJCC stage with recurrence after 3 years.^8^ However, this study did not include squamous cell carcinomas, was performed using the AJCC seventh edition staging system, and did not provide data on types of lung resections included. Similar to the current study, investigators examining other malignancy types have reported that high-risk features at the time of treatment are associated with greater differences between CS and actuarial survival estimates. For example, the calculated CS exceeded the actuarial survival for patients with resected cholangiocarcinoma and high-risk features, including larger tumor size and lymph node metastasis.^11^

An estimated 20% to 50% of patients with resected early-stage NSCLC will experience recurrence, most within the first 2 years.^23^, ^24^, ^25^, ^26^ It is likely that a combination of the molecular profile of the tumor and individual patient characteristics contribute to a patient's risk of recurrence.^14^^,^^16^^,^^17^^,^^26^, ^27^, ^28^ Accordingly, the cohort of patients that remain alive and disease free through the early postoperative years, when recurrence is most common, have a different prognosis than seen the initial cohort at the time of treatment. This is reflected in the difference between actuarial survival and CS estimates. CS analyses account for time survived after surgery and ultimately provide more accurate estimates of prognosis for patients from that time point onward.

The differential between actuarial and conditional OS was smaller than that between actuarial and conditional DFS. There may be several explanations for this observation. Despite not experiencing recurrence, patients with larger tumors or greater AJCC stage carried a higher comorbidity burden, including diabetes, CAD, and CHF. These comorbidities have the potential to affect OS without changing the risk of DFS.

As a patient accumulates years of survival, the relevance of certain prognostic factors present at the time of surgery changes. CS analysis is a powerful tool for estimating how prognosis changes in patients over time. These findings have important implications for clinical decision making and patient counseling. They may allow for individualized surveillance schedules and help alleviate patient anxiety as they survive for longer periods after surgery.

This study has several limitations. Its single institution retrospective nature decreases the external validity of our findings. Despite the large number of subjects and long-term follow-up, survival estimates beyond 5 years became limited owing to a smaller total study population. Validation within both North American and international populations would allow for greater generalization of our results.

## Conclusions

In patients with resected stage I-IIIA NSCLC, CS estimates remain greater than actuarial survival rates for both OS and DFS. Patients with characteristics most strongly associated with mortality and recurrence demonstrate the greatest difference between CS and actuarial survival. In addition, sex, tumor size, node positivity, and AJCC stage may have a diminishing prognostic effect as time passes. These findings suggest that CS analyses provide a quantitative representation of changes in patient prognosis after treatment that may be valuable to both patients and providers.

## Conflict of Interest Statement

The authors reported no conflicts of interest.

The *Journal* policy requires editors and reviewers to disclose conflicts of interest and to decline handling or reviewing manuscripts for which they may have a conflict of interest. The editors and reviewers of this article have no conflicts of interest.

## Figures and Tables

**Table E1:** Recurrences by year after surgery

Year after surgery	Recurrences
1	66
2	66
3	42
4	25
5	13
6	7
7	7
8	2
